# Telomere Length Is Not Related to Established Cardiovascular Risk Factors but Does Correlate with Red and White Blood Cell Counts in a German Blood Donor Population

**DOI:** 10.1371/journal.pone.0139308

**Published:** 2015-10-07

**Authors:** Bruno Neuner, Anna Lenfers, Reinhard Kelsch, Kathrin Jäger, Nina Brüggmann, Pim van der Harst, Michael Walter

**Affiliations:** 1 Department of Anaesthesiology and Intensive Care Medicine, Campus Virchow-Klinikum, Charité, University Medicine, Berlin, Germany; 2 Department of Anaesthesiology and Intensive Care Medicine, Campus Charité-Mitte, Charité, University Medicine, Berlin, Germany; 3 Department of Gynecology and Obstetrics, University Hospital Münster, Münster, Germany; 4 Institute of Transfusion Medicine, University Hospital Münster, Münster, Germany; 5 Institute of Laboratory Medicine, Clinical Chemistry and Pathobiochemistry, Campus Virchow-Klinikum, Charité, University Medicine, Berlin, Germany; 6 Labor Berlin, Charité Vivantes Services GmbH, Berlin, Germany; 7 Department of Cardiology, University Medical Center Groningen, University of Groningen, Groningen, The Netherlands; University of Louisville, UNITED STATES

## Abstract

Telomere length (TL) is considered a marker of biological aging and has been associated with the presence of various coronary risk factors in patients. Much less is known about the relationships between TL and classic coronary risk factors in other populations. We measured TL in peripheral blood leukocytes of 343 middle-aged blood donors (mean age 40.2 ± 12.4 years; 201 men, 142 women) using quantitative polymerase chain reaction. Median TL was 0.86 (range: 0.48–1.85) relative TL units. In linear regression analyses with natural log-transformed T to S ratio as the dependent variable, there was a significant association with age (per year: beta = -0.007, p<0.001) and sex (males vs. females: beta = 0.075, p = 0.007) with longer telomeres in men. After adjusting for these two variables, we observed no association of TL with classic coronary risk factors including cholesterol (p = 0.36), triglyceride (p = 0.09), HDL-cholesterol (p = 0.26), LDL-cholesterol (p = 0.36), smoking (p = 0.97), and personal (p = 0.46) or family history (p = 0.63) of cardiovascular disease. However, we did find a significant positive association with white (p = 0.011) and red blood cell count (p = 0.031), hemoglobin (p = 0.014) and hematocrit (p = 0.013); we also found a borderline positive association with thrombocytes (p = 0.074). Positive associations remained significant for hemoglobin (p = 0.017), hematocrit (p = 0.023), and leukocytes (p = 0.009) in a subgroup with no reported vascular disease; associations were of borderline significance for erythrocytes (p = 0.053) and thrombocytes (p = 0.088) in this subgroup. The data do not support the concept that classic coronary risk factors contribute to telomere attrition in a blood donor population. However, telomere attrition may be a marker for reduced proliferation reserve in hematopoietic progenitor cells.

## Introduction

Telomeres are DNA protein complexes at the ends of linear chromosomes in eukaryotes that serve a protective function during mitosis by stabilizing the chromosomal structure. Due to inherent characteristics of the replication machinery in eukaryotes, and the so-called end-replication problem, TL shortens with each cell division and can be viewed as a mitotic clock reflecting cellular turnover. Shortening to a critical length induces ‘replicative senescence’ via several mechanisms, the most important of which seems to be a DNA damage-like response. Replicative senescence includes replication arrest and may thus represent a tumor suppressor mechanism protecting cells from transformation. As a drawback, however, telomere shortening limits regenerative capacity. This fact has contributed to the development of the idea that TL can be seen as a surrogate marker (and possibly also direct propagator) of the aging process and a potential key to longevity. Furthermore, TL is being considered as a potential biomarker of age-related chronic cardiovascular diseases, including atherosclerosis and heart failure [1,2] because it reflects increased cellular stress and cell turnover in these diseases.

Indeed, several studies have shown an association between TL and coronary heart disease. Shorter TLs were found in coronary endothelium of diseased coronary arteries [3] but were also detectable in peripheral blood leukocytes of coronary heart disease patients [4–6]. Shorter TLs were found in aneurysm biopsies [7] and in peripheral blood leukocytes of patients with aortic aneurysms [8]. Reduced TLs were detected in leukocytes of hypertensive and diabetic patients with carotid artery disease [9,10] and in leukocytes of patients with chronic heart failure suffering from peripheral vascular disease [11].

Less clear is the relationship between classic coronary risk factors and TL in apparently healthy people. In the Framingham Study, hypertensive male subjects had shorter age-adjusted TL than normotensive controls [12]. The Cardiovascular Health Study, however, could not confirm these data in a mixed male and female cohort [6]. Leukocyte TL was also inversely related to body mass index and diabetes mellitus in some studies [10,13,14]. But these correlations were, if at all, only partially reproduced in other studies [15,16]. A dose-dependent effect of smoking on telomere attrition was observed in two studies [13,17] but was not found in two other studies [18,19]. In another study, no association between TL and lipid status or blood pressure was observed in a middle-aged population [20].

In this study, we measured TL in peripheral blood leukocytes of middle-aged blood donors and looked for associations between it and classic coronary risk factors and various so-called emerging risk factors that may indicate the onset of organ dysfunction. To measure TL, we used a high-end very precise PCR-based method.

## Materials and Methods

### Study design and determination of coronary risk factors

This study was approved by the Medical Ethical Committee Münster and all subjects provided written informed consent. Blood donors were between 18 and 70 years old, and all participants filled out an eligibility questionnaire disclosing any known health conditions or high-risk behaviour before each blood donation. The rules of the blood donation center exclude any individuals from blood donation who have a hemoglobin lower than 12.5 mg/dl in women and 13.0 mg/dl in men, or who are suffering from chronic diseases or infections from blood donation. Body weight and blood pressure were recorded. Additionally a number of laboratory routine parameters including cholesterol, triglycerides, glucose, creatinine, and blood cell counts were measured using autoanalyzers (Siemens, Roche). A total of 360 consecutively elected voluntary blood donors (210 men, 150 women) were eligible. We had TL measurements for 343 out of these 360 eligibles. The missing 17 values are due to lack of sufficient DNA amounts or DNA quality problems. Lifestyle factors, medical history, and demographics were recorded using standardized questionnaires. These included: smoking (current smoking or non-smoking), alcohol use, body weight, physical activity, diabetes, and blood pressure, as well as personal and family history of myocardial infarction, apoplexy, or other vascular disease. Alcohol use was classified using an alcohol-index with three categories: abstention; moderate drinking (less than three alcoholic drinks daily); and heavy drinking (three or more alcoholic drinks daily). Body mass index (BMI) was calculated as weight divided by height squared (kg/m^2^). Personal or family history of vascular disease was defined as the occurrence of myocardial infarction, apoplexy, or any other vascular disease either in the blood donor, their parents, siblings, and offspring, as reported by the donor in a questionnaire. The typical signs of peripheral arterial or venous disease were described in the questionnaire as aching, cramping, pain or swelling in the arms or legs, particularly when walking or exercising; feeling of heaviness in the legs, and pain or cramps in the calves. The PROCAM (Prospective Cardiovascular Münster Study) score was calculated using age, gender, diabetes mellitus (Y/N) or fasting blood glucose levels > 120mg/dl (Y/N), current nicotine consumption (Y/N), positive family history of cardiovascular disease (Y/N), systolic blood pressure in mmHg, LDL cholesterol, HDL cholesterol and triglycerides.

### Telomere length measurements and routine laboratory measurements

Mean TL was measured with the recently modified QPCR protocol using a single well strategy to measure to the telomere (T) and single reference (S) signal [21]. The ratio of telomere and reference gene content (T/S ratio) is a relative measure of TL and is expressed in arbitrary units. A reference DNA standard (5.2 to 60 ng), a no template (water) control, and a positive/maximum control (human leukemia cell line (1301) kindly provided by dr. Cesaro, IST, Genova) were run in duplicate on each plate on a Bio-Rad CFX384 real-time C1000 thermal cycler. Samples were run in triplicate on different plates. The final concentrations of reagents in the PCR were 1U Titanium Taq DNA polymerase with the provided Titanium Taq PCR buffer, 0.75xSYBR Green I (Sigma), 0.2 mM of each dNTP, 1 mM DTT, 1M betaine, 900nM of each primer. The primers used were; telomere (T), telg, 5’-ACACTAAGGTTTGGGTTTGGGTTTGGGTTTGGGTTAGTGT–3’ and telc, 5’-TGTTAGGTATCCCTATCCCTATCCCTATCCCTATCCCTAACA–3’, that generate a short, fixed-length product. The primers of the Single copy gene (S) used were albu: 5’- cggcggcgggcggcgcgggctgggcggAAATGCTGCACAGAATCCTTG–3’ albd: 5’-gcccggcccgccgcgcccgtcccgccgGAAAAGCATGGTCGCCTGTT–3’. Lower case letters of the albumin primers are non-template 5’ tag sequences that confer a high melting temperature on resulting PCR product allowing monochrome multiplexing. The thermal cycling profile was 15 min at 95°C; 2 cycles of 15 s at 94°C, 15 s at 49°C; 5 cycles of 15 s at 94°C, 15 s at 66°C; 32 cycles of 15 s at 94°C, 10 s at 60°C, 15 s at 72°C with T signal acquisition, 10 s at 85°C, and 15 s at 89°C with S signal acquisition. For further explanation and details, see reference [21]. Reproducibility data was obtained for 343 independent subjects and good agreement between T/S ratios was observed (R^2^ = 0.99, P<0.0001, inter-run CV 3.9%).

### Statistical methods

**Fig 1:**
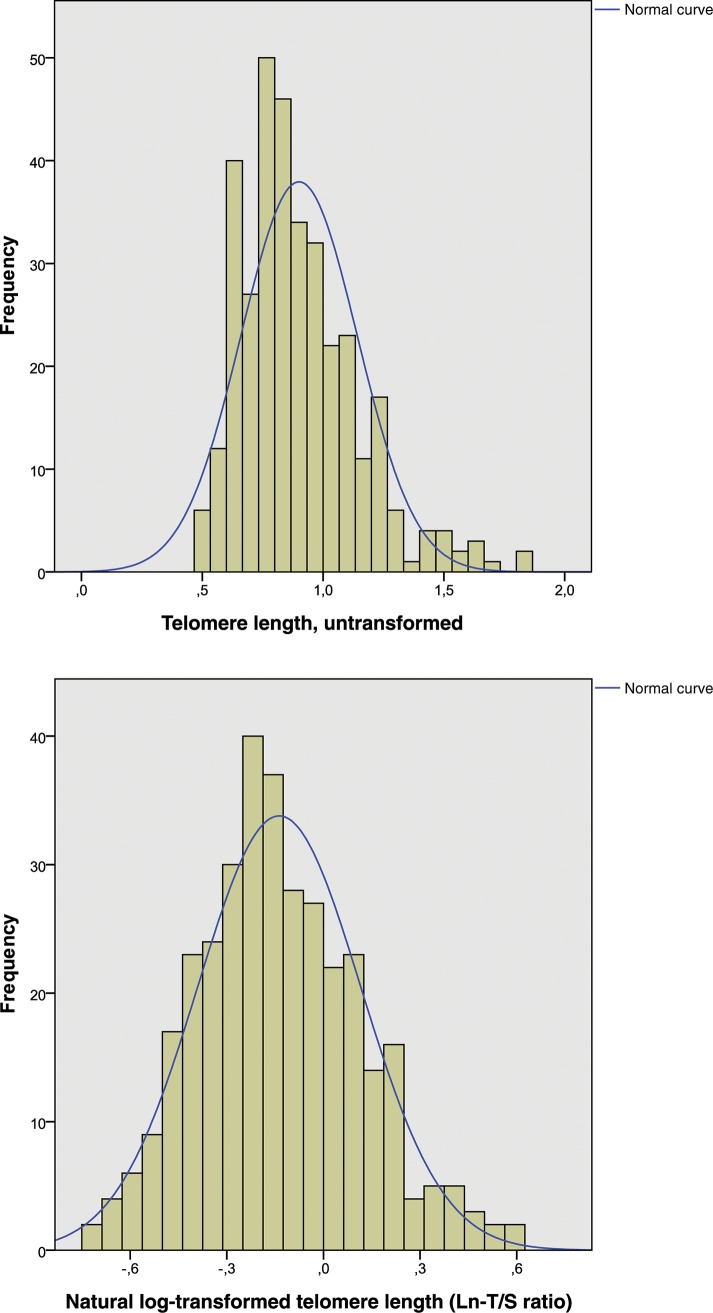
Telomere length (untransformed) in all patients, n = 343 (A); Telomere length (natural log-transformed) in all patients, n = 343 (B). T/S was non-normally distributed (A). A natural log-transformation was performed to obtain a near-normal distribution (B).

Normally distributed continuous variables were presented as mean ± SD, and non-normally distributed continuous variables were presented as median (range). As the ratio of telomere and reference gene content (T/S ratio) showed a deviation from a normal distribution (Kolmogorov–Smirnov test for normal distribution: p < 0.001), natural log-transformed T to S ratios (Ln-T/S ratio) were used as surrogate parameters for TL (Kolmogorov–Smirnov test for normal distribution: p = 0.063, as shown in Fig 1). Dichotomous variables were presented as total numbers and percentages. To test for differences between two independent subgroups (males and females, Table 1), chi-square tests respectively Fisher’s exact tests (when n ≤ 5) were used for dichotomous variables. To test for differences in continuous variables with normal and non-normal distributions, we used t-tests respectively Mann-Whitney tests. The correlation between TL and age was evaluated using Pearson correlation coefficient. Associations between TL (dependent variable) and cardiovascular risk factors, lifestyle characteristics, and other parameters were analyzed using linear regression analyses. Results are shown for unadjusted and age- and gender-adjusted analyses (Table 2). A p *<* 0.05 (2 sided) was considered statistically significant. All statistical calculations were performed using the Statistical Package for the Social Sciences (SPSS v22.0).

**Table 1:** Telomere length and baseline characteristics in all patients and stratified by gender, n = 343.

Parameter	Total(N = 343)	Men(N = 201)	Women(N = 142)	p Value ^#^
Age in years (min–max)	40.2 ± 12.4(18–71)	39.3 ± 11.7(18–71)	41.6 ± 13.3(19–70)	0.10
T to S ratio (qPCR), untransformed	0.86 (0.48–1.85)	0.88 (0.49–1.85)	0.81 (0.48–1.64)	0.007 ^MWU-test^
T to S ratio (qPCR), natural log-transformed	-0.14 ± 0.25	-0.11 ± 0.26	-0.18 ± 0.24	0.007
Smoking, n (%) (n = 342)	76 (22.%)	52 (26.0%)	24 (16.9%)	0.046
Pack-years in smokers (n = 76)	12.0 ± 9.5	12.6 ± 9.4	10.7 ± 9.9	0.42
Body mass index, kg m^−2^ (n = 340)	24.5 (18.4–41.4)	25.1 (19.2–31.9)	23.5 (18.4–41.4)	< 0.001 ^MWU-test^
Obesity (BMI > 30 kg m^−2^), n (%) (n = 340)	33 (9.7%)	20 (10.1%)	13 (9.2%)	0.77
Glucose, mmol/L (n = 338)	82 (37–332)	84 (37–332)	80 (39–200)	0.15 ^MWU-test^
Hypertension, n (%)	4 (1.2%)	1 (0.5%)	3 (2.1%)	0.31^$^
Systolic blood pressure, mmHg	125.0 ± 9.0	126.8 ± 9.4	122.4 ± 7.6	<0.001
Diastolic blood pressure, mmHg	79.3 ± 4.8	80.2 ± 4.4	78.1 ± 5.0	<0.001
Cholesterol, mmol/L (n = 338)	196 ± 38	194 ± 35	200 ± 41	0.18
LDL cholesterol, mmol/L (n = 339)	114 ± 32	115 ± 32	113 ± 32	0.56
Hypercholesterolemia (LDL > 130 mmol/L), n (%) (n = 339)	96 (2.38%)	65 (32.5%)	31 (22.3%)	0.040
HDL cholesterol, mmol/L(n = 339)	57 ± 15	52 ± 12	64 ± 16	<0.001
Triglycerides, mmol/L (n = 338)	115 (10–432)	121 (10–432)	106 (36–370)	0.006 ^MWU-test^
Triglycerides > 150 mmol/L + HDL < 50 mmol/L (♀) resp. < 40 mmol/L (♂), n (%) (n = 338)	35 (10.4%)	21 (10.6%)	14 (10.1%)	0.89
Metabolic Syndrome^#^, n (%) (n = 339)	94 (27.7%)	49 (24.5%)	45 (32.4%)	0.11
Fibrinogen, μmol/L (n = 333)	269 (80–694)	260.5 (97–578)	299 (80–694)	< 0.001 ^MWU-test^
Hyperfibrinogenemia (fibrinogen > 350 μmol/L), n (%) (n = 333)	52 (15.6%)	18 (9.2%)	34 (24.8%)	< 0.001
Creatinine, μmol/L ^†^ (n = 337)	0.87 ± 0.16	0.94 ± 0.13	0.76 ± 0.12	< 0.001
GOT, U/L (n = 338)	24 (14–148)	26 (16–137)	22 (14–148)	< 0.001 ^MWU-test^
GPT, U/L (n = 338)	18 (2–266)	21 (2–150)	14 (4–266)	<0.001 ^MWU-test^
Hemoglobin, g/dL (n = 317)	14.2 ± 1.3	14.9 ± 1.1	13.3 ± 0.9	<0.001
Hematocrit, % (n = 316)	40.4 ± 3.2	41.9 ± 2.9	38.3 ± 2.4	<0.001
Erythrocytes, 10^12^/L (n = 316)	4.6 ± 0.4	4.8 ± 0.4	4.4 ± 0.3	<0.001
MCV, 10^−15^ L (n = 317)	87.0 ± 4.0	87.0 ± 3.9	87.1 ± 4.1	0.87
MCH, pg (n = 316)	30.8 (2.5–35.1)	31.1 (24.9–35.1)	30.4 (2.5–34.2)	0.002 ^MWU-test^
MCHC, g/dL (n = 317)	35.2 (30.8–345.6)	35.6 (32.9–345.6)	35.0 (30.8–36.8)	<0.001 ^MWU-test^
Leukocytes, 10^9^/L (n = 317)	5.7 ± 1.4	5.5 ± 1.2	5.9 ± 1.5	0.051
Thrombocytes, 10^9^/L (n = 317)	243.1 ± 57.1	228.9 ± 50.1	263.0.0 ± 60.4	<0.001
Vascular disease, %	19 (5.5%)	4 (2.0%)	15 (10.6)	0.001
Positive family history of vascular disease, n (%) (n = 342)	118 (34.5%)	54 (27.0%)	64 (45.1%)	0.001

Count data presented as n(%) and continuous data given as mean ± SD or median (range), respectively

# independent-samples t test respectively Mann-Whitney-U-Test (MWU-test) or the χ^2^ test

† one outlier removed

$ = Fisher’s Exact Test

BMI = Body mass index

LDL = low density lipoprotein

HDL = high density lipoprotein

GOT = Glutamat-Oxalacetat-Transaminase

GPT = Glutamat-Pyruvat-Transaminase

MCV = Mean corpuscular volume

MCH = Mean corpuscular hemoglobin

MCHC = Mean corpuscular hemoglobin concentration

# = Hyperfibrinogenemia and/or obesity and/or triglycerides > 150 mmol/L combined with HDL < 50 mmol/L (♀) resp. < 40 mmol/L (♂).

**Table 2:** Influence of risk factors on telomere length (natural log-transformed T/S ratio), results of linear regression models, n = 343.

Independent parameters	unadjusted			adjusted#		
	beta_x_	95%-CI	p-value	beta_x_	95%-CI	p-value
Age, per year	-0.007	-0.009 to -0.005	< 0.001	-	-	-
Male vs. female gender	0.075	0.021 to 0.129	0.007	-	-	-
Smoking, yes vs. no	0.027	-0.038 to 0.092	0.41	0.001	-0.060 to 0.062	0.97
Pack-years, per year (in smokers)	0.008	-0.021 to 0.037	0.53	-0.003	-0.030 to 0.025	0.85
Body mass index, per unit (kg/m^2^)	-0.007	-0.014 to 0.000	0.05	-0.004	-0.011 to 0.003	0.23
Obesity (BMI > 30 kg m^−2^), yes vs. no	-0.024	-0.115 to 0.067	0.60	-0.009	-0.094 to 0.076	0.84
Glucose, per 10 mmol/L	-0.012	-0.023 to -0.001	0.033	-0.005	-0.016 to 0.006	0.36
Systolic blood pressure, per 10 mmHg	0.002	-0.028 to 0.032	0.92	0.003	-0.026 to 0.033	0.82
Diastolic blood pressure, per 10 mmHg	-0.028	-0.084 to 0.028	0.33	-0.022	-0.076 to 0.033	0.43
Cholesterol, per 10 mmol/L	-0.014	-0.021 to– 0.007	< 0.001	-0.004	-0.011 to 0.004	0.36
LDL cholesterol, per 10 mmol/L	-0.015	- 0.023 to -0.006	0.001	-0.004	-0.013 to 0.005	0.36
Hypercholesterolemia (LDL > 130 mmol/L), yes vs. no	-0.071	-0.130 to -0.011	0.020	-0.015	-0.075 to 0.045	0.62
HDL cholesterol, per 10 mmol/L	-0.017	-0.034 to 0.001	0.065	-0.010	-0.028 to 0.008	0.26
Triglycerides, per 10 mmol/L	0.002	-0.002 to 0.005	0.44	0.003	0.000 to 0.007	0.086
Triglycerides > 150 mmol/L + HDL < 50 mmol/L (♀) resp. < 40 mmol/L (♂), yes vs. no	0.020	-0.070 to 0.109	0.67	0.038	-0.045 to 0.121	0.37
Metabolic Syndrome^##^, yes vs. no	-0.048	-0.108 to 0.012	0.12	0.012	-0.046 to 0.071	0.68
Fibrinogen, per 10 μmol/L	-0.005	-0.009 to– 0.002	0.002	-0.002	-0.006 to 0.001	0.22
Hyperfibrinogenemia (fibrinogen > 350 μmol/L), yes vs. no	-0.064	-0.138 to 0.010	0.09	0.014	-0.060 to 0.088	0.72
Creatinine, per 10 μmol/L^†^	0.012	-0.212 to 0.236	0.92	-0.024	-0.234 to 0.186	0.82
GOT, per 10 U/L	-0.001	-0.026 to 0.024	0.94	-0.009	-0.033 to 0.014	0.44
GPT, per 10 U/L	-0.011	-0.025 to 0.003	0.13	-0.014	-0.028 to -0.001	**0.039**
Hemoglobin per 10 g/dL	0.378	0.161 to 0.595	0.001	0.322	0.067 to 0.577	**0.014**
Hematocrit, per %	0.015	0.006 to 0.024	0.001	0.012	0.003 to 0.022	**0.013**
Erythrocytes, per 10^12^/L	0.128	0.063 to 0.193	< 0.001	0.078	0.007 to 0.149	**0.031**
MCV, per 10 fL	-0.058	-0.129 to 0.013	0.11	0.005	-0.064 to 0.073	0.89
MCH, per 10 pg	-0.033	-0.151 to 0.085	0.58	0.029	-0.085 to 0.143	0.62
MCHC, per 10 g/dL	0.004	-0.012 to 0.020	0.64	-0.001	-0.016 to 0.015	0.95
Leukocytes, per 10^9^/L	0.013	-0.007 to 0.034	0.21	0.025	0.006 to 0.044	**0.011**
Thrombocytes, per 10^12^/L	0.177	-0.317 to 0.672	0.48	0.436	-0.043 to 0.914	0.074
Vascular disease, yes vs. no	-0.136	-0.253 to -0.019	0.023	-0.043	-0.156 to 0.071	0.46
Positive family history of vascular disease, yes vs. no	-0.059	-0.115 to -0.003	0.041	0.014	-0.043 to 0.071	0.63

#: adjustments for age and sex

CI = confidence interval

† = one outlier removed before regression analyses

BMI = Body mass index

LDL = low density lipoprotein

HDL = high density lipoprotein

GOT = Glutamat-Oxalacetat-Transaminase

GPT = Glutamat-Pyruvat-Transaminase

MCV = Mean corpuscular volume

MCH = Mean corpuscular hemoglobin

MCHC = Mean corpuscular hemoglobin concentration

## = Hyperfibrinogenemia and/or obesity and/or triglycerides > 150 mmol/L combined with HDL < 50 mmol/L (♀) resp. < 40 mmol/L (♂).

## Results

### Population characteristics and relationship to telomere length

Baseline demographics, vascular risk factors, laboratory parameters, and T to S ratio in men and women of the study population, i.e. patients with complete data on TL (N = 343), are presented in Table 1. TL was slightly and significantly longer in men than in women (0.88 (95% confidence interval (0.49–1.85)) vs. 0.81 ((95% confidence interval (0.48–1.64)); p = 0.007). Women had a slightly lower systolic (122.6 ± 7.6 mmHg vs. 126.7 ± 9.4 mmHg; P<0.001) and diastolic (78.1 ± 5.0 mmHg vs. 80.2 ± 4.4 mmHg; P<0.001) blood pressure, a higher HDL-cholesterol level (64 ± 16 mmol/L vs. 52 ± 12 mmol/L; p<0.001), a higher fibrinogen concentration (299 (80–694) vs. 260.5 (97–578) μmol/L; p<0.001, Mann-Whitney-U-test) as well as a higher prevalence of vascular disease (10.6% vs. 2.0%), P = 0.001 and family history of vascular disease (44.0% vs. 27.1%; p<0.001). The mean values for erythrocytes, hematocrit and hemoglobin were higher in men (all p< 0.001) and were within the reference values for men and women, respectively.

**Fig 2:**
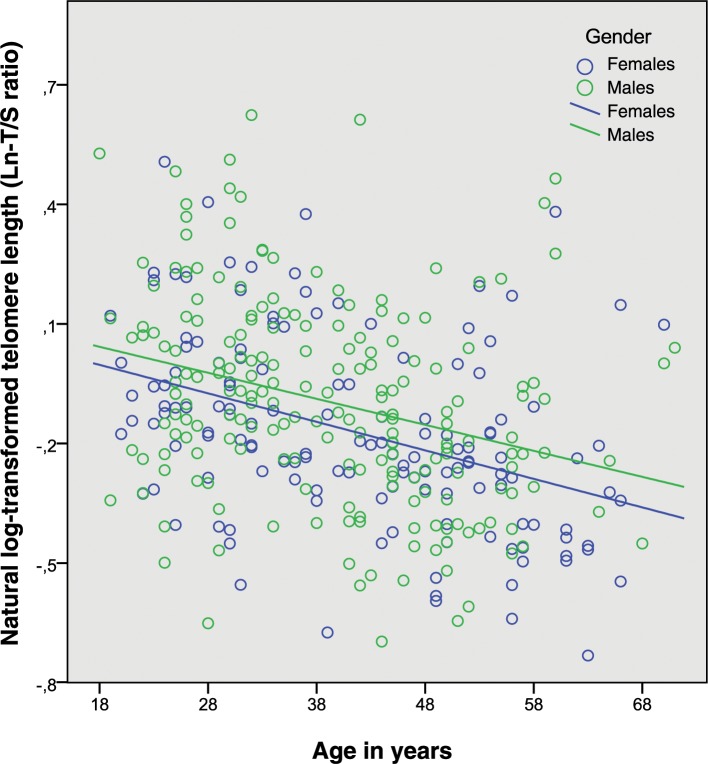
Age and natural log-transformed telomere length, males and females, n = 343. The association of TL with age, visualized by the regression lines for men (blue circles) and women (green circles), respectively.

As shown in Fig 2, we observed a highly significant inverse association of the T/S ratio (as surrogate of TL) and chronological age (r = -0.345, P<0.001). Accordingly age explained approximately 12% of the variability of T/S (r^2^ = 0.1191).

### Cardiovascular risk and telomere length

Other studies have shown an accelerated telomere shortening being related to coronary risk factors, including obesity, smoking and diabetes mellitus. These effects have been presumed to be mediated through the increased oxidative stress caused by these factors. We were therefore interested in the relationship between the Ln-T/S ratio (as surrogate marker of TL) and these risk factors as well as selected laboratory values.

As shown in Table 2, TL was associated with age (p<0.001) and sex (p = 0.007). Therefore analyses were adjusted for these covariates (right column in Table 2). In unadjusted models (left column in Table 2), glucose, total cholesterol and LDL cholesterol, and fibrinogen were associated with TL. These associations were not seen after adjustments for age and sex. However several blood cell counts showed significant association with TL even after adjustments: this was true of red blood cell counts (per 10^12^/L: Beta: 0.078 (95%-CI (0.007 to 0.149); p = 0.031), hemoglobin (per 10 g/dL: Beta: 0.322 (95%-CI (0.067 to 0.577)), p = 0.014) and hematocrit values (per %, Beta: 0.012 (95%-CI (0.003 to 0.022)), p = 0.013). A borderline significance was observed for an association between TL and thrombocytes (per 10^12^/L: Beta 0.436 (95%-CI (-0.043 to 0.914)), p = 0.074) and a significant association with white blood cell counts (per 10^9^/L: Beta: 0.025 (95%-CI (0.006 to 0.044)), p = 0.011. Additionally, one liver enzyme, GPT, was significantly inversely associated with TL: per 10 U/L: Beta -0.014 (95%-CI (-0.028 to -0.001)), p = 0.039).

In a subgroup analysis, we examined the data of all blood donors with no reported vascular disease (n = 324). As shown in Table 3, results were very similar in this group of apparently healthy volunteers. Again, in unadjusted models, TL was associated with glucose, total cholesterol, HDL and LDL cholesterol, fibrinogen and several blood cell counts. However, after adjustment for age and sex, significant associations remained between TL and haemoglobin (per 10 g/dL: Beta: 0.320 (95%-CI (0.057 to 0.584)), p = 0.017) and hematocrit (per %: Beta: 0.012 (95%-CI (0.002 to 0.022)), p = 0.023). A borderline significance was observed for an association of TL and red blood cell counts (per 10^12^/L: Beta: 0.072 (95%-CI (-0.001 to 0.146)), p = 0.053). While there was no association in unadjusted models, adjustment for age and sex led to a borderline association of TL with thrombocytes (per 10^12^/L. Beta 0.432 (95%-CI (-0.065 to 0.930)), p = 0.088) as well as to a significant association with white blood cell counts (per 10^9^/L: Beta: 0.027 (95%-CI (0.007 to 0.047)), p = 0.009). Again, an inverse association of TL and GPT appeared but only at a level of borderline significance: per 10 U/L: Beta: -0.013 (95%-CI (-0.028 to 0.001)), p = 0.076.

**Table 3:** Influence of risk factors on telomere length (natural log-transformed T/S ratio), results of linear regression models in patients without vascular disease, n = 324.

Independent parameters	unadjusted			adjusted^#^		
	beta_x_	95%-CI	p-value	beta_x_	95%-CI	p-value
Age, per year	-0.007	-0.009 to -0.004	< 0.001	-	-	-
Male vs. female gender	0.064	0.008 to 0.120	0.003	-	-	-
Smoking, yes vs. no	0.020	-0,046 to 0.085	0.56	-0.003	-0.065 to 0.060	0.94
Pack-years, per year (in smokers)	0.007	-0.022 to 0.036	0.63	-0.002	-0.030 to 0.025	0.86
Body mass index, per unit (kg/m^2^)	0.006	-0.013 to 0.001	0.11	-0.004	-0.011 to 0.003	0.28
Obesity (BMI > 30 kg m^−2^), yes vs. no	0.004	-0.093 to 0.102	0.93	0.007	-0.085 to 0.099	0.88
Glucose, per 10 mmol/L	-0.012	-0.024 to -0.001	0.033	-0.005	-0.017 to 0.006	0.34
Systolic blood pressure, per 10 mmHg	0.003	-0.028 to 0.033	0.86	0.004	-0.025 to 0.034	0.77
Diastolic blood pressure, per 10 mmHg	-0.021	-0.079 to 0.038	0.49	-0.014	-0.071 to 0.043	0.64
Cholesterol, per 10 mmol/L	-0.012	-0.020 to– 0.005	0.001	-0.003	-0.011 to 0.005	0.49
LDL cholesterol, per 10 mmol/L	-0.012	- 0.021 to -0.004	0.006	-0.002	-0.012 to 0.007	0.60
Hypercholesterolemia (LDL > 130 mmol/L), yes vs. no	-0.056	-0.117 to 0.006	0.078	-0.005	-0.067 to 0.058	0.88
HDL cholesterol, per 10 mmol/L	-0.019	-0.037 to -0.001	0.044	-0.013	-0.032 to 0.006	0.17
Triglycerides, per 10 mmol/L	0.002	-0.002 to 0.006	0.37	0.003	-0.001 to 0.007	0.090
Triglycerides > 150 mmol/L + HDL < 50 mmol/L (♀) resp. < 40 mmol/L (♂), yes vs. no	0.048	-0.046 to 0.142	0.31	0.062	-0.026 to 0.151	0.17
Metabolic Syndrome^##^, yes vs. no	-0.029	-0.092 to 0.034	0.37	0.024	-0.037 to 0.085	0.44
Fibrinogen, per 10 μmol/L	-0.005	-0.008 to– 0.001	0.007	-0.002	-0.005 to 0.002	0.38
Hyperfibrinogenemia (fibrinogen > 350 μmol/L), yes vs. no	-0.056	-0.133 to 0.021	0.16	0.018	-0.060 to 0.095	0.65
Creatinine, per 10 μmol/L^†^	-0.001	-0.224 to 0.223	0.99	-0.027	-0.239 to 0.185	0.80
GOT, per 10 U/L	-0.002	-0.027 to 0.024	0.90	-0.009	-0.033 to 0.016	0.49
GPT, per 10 U/L	-0.010	-0.025 to 0.005	0.20	-0.013	-0.028 to 0.001	0.076
Hemoglobin, per 10 g/dL	0.345	0.124 to 0.566	0.002	0.320	0.057 to 0.584	**0.017**
Hematocrit, per %	0.014	0.005 to 0.023	0.003	0.012	0.002 to 0.022	**0.023**
Erythrocytes, per 10^12^/L	0.114	0.048 to 0.181	0.001	0.072	-0.001 to 0.146	0.053
MCV, per 10 fL	-0.048	-0.121 to 0.024	0.19	0.007	-0.064 to 0.078	0.84
MCH, per 10 pg	-0.020	-0.139 to 0.098	0.74	0.037	-0.079 to 0.153	0.53
MCHC, per 10 g/dL	0.004	-0.013 to 0.020	0.67	0.000	-0.015 to 0.015	0.97
Leukocytes, per 10^9^/L	0.015	-0.006 to 0.036	0.17	0.027	0.007 to 0.047	**0.009**
Thrombocytes, per 10^12^/L	0.237	-0.272 to 0.745	0.36	0.432	-0.065 to 0.930	0.088
Positive family history of vascular disease, yes vs. no	-0.038	-0.097 to -0.021	0.21	0.022	-0.037 to 0.081	0.46

#: adjustments for age and sex

CI = confidence interval

† = one outlier removed before regression analyses

BMI = Body mass index

LDL = low density lipoprotein

HDL = high density lipoprotein

GOT = Glutamat-Oxalacetat-Transaminase

GPT = Glutamat-Pyruvat-Transaminase

MCV = Mean corpuscular volume

MCH = Mean corpuscular hemoglobin

MCHC = Mean corpuscular hemoglobin concentration

## = Hyperfibrinogenemia and/or obesity and/or triglycerides > 150 mmol/L combined with HDL < 50 mmol/L (♀) resp. < 40 mmol/L (♂).

## Discussion

The observed inverse association between TL and biological age demonstrated here has also been described in many other studies and confirms the validity of our measurements [22–24].

TL was slightly but significantly longer in men than in women. This result was surprising because in the majority of other studies on the subject, either TL was found to be slightly longer in women than in men or no difference was observed [22–24]. The women of this cohort had a higher than average incidence of familial vascular disease, which may point to a higher genetic risk that may have contributed to accelerated telomere attrition and thus higher cardiovascular disease risk. However, the gender difference was very similar in the subgroup of blood donors without vascular disease (whole group: Beta = 0.075; p = 0.007; subgroup: Beta = 0.064; p = 0.003). Moreover, it has been shown that the gender difference described in most studies (TL female > TL male) is smaller at older ages and that smoking and meat consumption may contribute to these differences [25]. In this context, the relatively low prevalence of smoking (in both men and women) in our middle-aged population is of interest and may have, at least partly, contributed to this result. Ethnic diversity between populations or methodological differences (Southern blot versus PCR for TL analysis) might also explain the observed differences. For example, PCR-based methods are less sensitive to possible epigenetic differences between men and women that may influence recognition sites for restriction endonucleases used in other methods. It is noteworthy that a different study that also used a PCR-based methodology for measuring TL recently observed longer telomeres in men in another German cohort of older individuals [26]. On the other hand, not all studies that used PCR-based methodology found longer telomeres in men [24]. Thus methodology is likely not the only explanation for the observed gender difference.

Progressive telomere shortening has been observed in vascular regions susceptible to atherosclerosis [27], and may be directly involved in senescence induction. This in turn may, as a primary abnormality, enhance atherogenesis [28]. Moreover, the chronic systemic inflammation in atherogenesis may secondarily lead to increased cell turnover and telomere attrition. In this study, we did not find a significant association of TL and the occurrence of well-established cardiovascular risk factors, after adjusting for age and sex. The found associations of TL with cardiovascular events, cholesterol, and glucose before adjustments for age and sex are likely caused by age-dependencies and the slightly higher cardiovascular disease risk women in this cohort demonstrated overall.

Shorter telomeres have been found to be associated with coronary heart disease in many studies [3–11]. However, much less is known about the relationship between classic coronary risk factors and TL in apparently healthy people. In accordance with the data described in this study, Bekaert and co-workers did not find associations between TL and lipid status or blood pressure in a middle-aged population [20]. In other studies, no relationship has been found between TL and hypertension [6], body mass index [18,19] or smoking behaviour [18,19] in apparently healthy individuals. In the Bekaert study, shorter TL was associated with increased levels of inflammation and oxidative stress markers. In our study, we did not find an association with fibrinogen or combined hyperlipidemia with HDL deficiency (as a possible surrogate for enhanced oxidative stress). We did not however examine direct oxidative stress markers such as interleukin 6, C-reactive protein or oxidized LDL. Interestingly, Brouilette and co-workers found shorter TL in 45 healthy offspring of subjects with coronary artery disease compared to 59 offspring from families without such a history [29]. In contrast, our study found no such relationship between TL and positive family history of vascular disease after adjusting for age and sex. These data are in accordance with the Asklepios study, in which no shorter telomere length could be found in healthy subjects with a family history of cardiovascular disease [30].

At least three possible explanations can account for discrepant results in apparently healthy individuals versus atherosclerotic patients. First, the power of this and other studies with negative results might be too low to detect minor differences. Second, the telomere attrition may provide an independent mechanism for the development of cardiovascular conditions and is not a reflection of the presence of established coronary risk factors. In this scenario the coincidence of telomere attrition and prevalence of other risk factors would not reflect a causal relationship. Third, telomere attrition happens at a relatively late event in chronic stress response, perhaps making is as yet undetectable in our healthy subject group.

Apart from classic coronary risk factors, we also selected emerging risk factors including creatinine, transaminases and blood cell counts to explore possible connections with TL. We found that TL was positively associated with erythrocyte and leukocyte count, haematocrit, and hemoglobin. A borderline association was found between TL and thrombocytes (Table 2). Altogether these data suggest that telomere attrition may be a marker for reduced global cellular reserve in this population.

Recent reports suggested an association between TL and blood cell counts. Anemia was found to be associated with shorter leukocyte TL in patients with heart failure [31]. De Meyer et al. found a positive association of TL and red blood cell count but no significant association of TL and haematocrit, haemoglobin or leukocytes in middle-aged subjects in the Asklepios study [32]. In contrast to our study, these authors found shorter TLs to be associated with increased MCV. In the Dallas Heart study, Kozlitina et al. also found a positive association of TL with red blood cell count and a negative association of TL and MCV, using a multiple regression model in a multi-ethnic population [33].

Conflicting results have similarly been described for leukocytes. Gutmajster and colleagues have seen an association of white blood cell count and TL in elderly Caucasians [34] and Aulinas et al. have seen such a correlation in patients with hypercortisolism [35]. By contrast, Satoh and co-workers were unable to show an association between TL and leukocyte count [36].

The origin of these differences might be rooted in varying study populations, ethnic diversity, methodological differences, a limited number of study participants, and lack of adjustment for possible modifiers of hematological blood values (such as thyroid stimulating hormone, folate, vitamin B12 and erythropoietin levels).

This study has some limitations. First, we cannot entirely exclude the possibility that blood donations made previous to the beginning of our study may have influenced the results, particularly regarding blood cells. It is unlikely, however, that this is the only explanation for our findings. Anemic individuals were excluded from blood donation and the relationship between TL and red or white blood cells (despite the described conflicting results) have previously been seen in other populations. Second, the cohort is heterogeneous and cardiovascular risk factors are unequally distributed among men and women. On the other hand, we observed very similar results in the subgroup without vascular disease. Overall, data are plausible insofar as we found significant associations of TL with both major cell lines, a borderline association with the megakaryocytic cell line, and associations with hemoglobin and hematocrit but no association with any of the established cardiovascular risk factors.

In summary, the data described here do not support the concept that coronary risk factors resulted in increased cell turnover and telomere attrition in a blood donor population. Whether TL could serve as a global biomarker of vascular risk, or if it may play a role as an independent risk factor remains to be determined in prospective studies. Telomere attrition may be a marker for reduced global cellular proliferation reserve.

## Supporting Information

S1 Table Raw data of all study participants. (XLS)
